# Pluggers for “Annealed Gold.”

**Published:** 1857-06

**Authors:** Louis Jack


### PLUGGERS FOR "ANNEALED GOLD."

BY LOUIS JACK, D. D. S.

In the use of "annealed gold," nothing is of more importance, probably, than the possession of suitable plugging instruments. There is need of some attention being paid to this subject, as it has seldom been treated of publicly with the fullness which its importance requires.

At this time I propose to explain in minute detail, a method which will enable any one of common ingenuity to secure for himself, points possessing the necessary qualities, without calling in the aid of the instrument maker.

I have no doubt many have been attracted by the descriptions given of the manner of using "annealed gold foil;" have attempted to follow them, and have failed to secure satisfactory results. This may be attributable, in the main, to the application of improper instruments. Those who have practised in this way, know, that without points of the best character and of appropriate forms, but poor results can be obtained. Now, in order to secure the perfect adhesion of each successive layer of gold with the least difficulty, a surface should be produced and kept up that is covered with pits and points, both of which should be acute. To secure this, it is necessary that the points on the end of the pluggers, whether they have a single one or are composed of a dozen, should be absolutely sharp, and the depressions between the points likewise have the same acuteness. If either point or pit, or both, are blunt, a corresponding surface will be given to the gold, which will not favor near so well the adhesion of the pieces as when sharper points are used. A pellet of annealed gold may, indeed, be easily made to adhere inseparably to a clean, smooth, unpolished surface of the same material; but it requires to be held in place at one point while another part is being condensed. In a tooth this would be a troublesome matter. The serration of the points is intended to overcome this difficulty.

It is found very convenient in practice to slightly attach a pellet in the middle of the filling, and then to drag pieces from it to fill up along the edges of the cavity. This is attended with much advantage. Anything that will increase the facility of performing this part of the operation of plugging teeth is certainly important, and the intelligent dentist will avail himself of anything, however slight it may be, that will aid him in the least. To those who have attempted to use gold in this manner, the tendency of the part of a pellet, first attached, to draw loose, while the remainder is being condensed, is well understood. To obviate this, it is necessary to increase the resistance by increasing the number and acuteness of the points.

I will now enter into a description of a method to completely secure the surface required, which occurred to me, and which has given me and others so much satisfaction as to constrain me to make a more public notice of this matter. Trifling as it is, to those who will fairly try it, it will be found very useful, and will save much time. This improvement consists simply in drawing the point backwards and forwards upon the heavy cuts of a Stubs file; thus giving to the instrument teeth as sharp as those of the file. The number of points in each plugger may vary from two up to twenty or thirty, all of which, in different classes of cavities, and under certain circumstances, will be required.

To make two, three, or more points in a line, the end of the instrument should first be hammered flat—and this should never be neglected in the manufacture of any instrument, as it adds greatly to the strength and hardness of the steel—then filed nearly to an edge, and, while straight, made smooth upon the Arkansas stone. The next step is to make the points. The instrument is held firmly by the fingers in the same position as the pen in writing, and rubbed lightly forwards and backwards upon the file, as before mentioned, commencing on one side somewhat, and turning the file or raising the handle of the instrument, until the teeth are marked upon the end, so that in turning it over to cut the other side, it can readily be adapted to the teeth of the file, in order to secure exact opposition of the cuts; a failure to effect this would render the points defective. The instrument should now be struck into the end of a block of hard wood to rub off the ragged corners, termed "wiring;" then, by very light rubbing upon the file and cleaning on the wood, the point will be ready for bending and finishing. It is quite important that this process should be neatly done, and the wiring well removed, else the gold may be dragged.

To make a square-ended plugger, having four points, which, by the way will be found to be one of the most useful forms, it is necessary, after reducing the steel small and square, to make two shallow cuts, intersecting each other at right angles, with a small knife-edge file; then dressing the steel quite close to the cuts. It can now be transferred to the flat surface of a file, and by the means of these lines, be readily adapted to one of the teeth, and the points completed much in the same way as described above. The cuts between the points will then be found quite sharp at the bottoms, which the best knife-edge files will not accomplish.

Another very useful form is composed of six and eight points, in two parallel rows. The steel is hammered flat, filed to twice the thickness of a wedge-shaped or line point, a longitudinal groove is made on the end, then dressed down on each side until two sharp wedge points are formed. It is then made smooth on the side, transferred to the file, and completed as directed for the single wedge shape. In this way, points can be made of such sharpness and fineness as cannot be formed in any other manner.

This will be found an excellent process to follow in making condensers. The objections to long, sharp points in this class of instruments, every one is aware of who uses them; the labor of removing their marks often requiring considerable time. In this way, by a little care, two sets of lines may be made at right angles to each other; the points so formed having the utmost sharpness, and yet they will be so short as not to impair the surface of the filling. In cutting the cross lines the instrument must be pressed slightly with a steady hand, else the first set will be injured or obliterated. This may be performed, however, sufficiently well with an ordinary thin-edged file. As many as sixty well-defined points may be given to a surface one-sixteenth of an inch square.

It may not be out of the way, now that we have directed the formation of the points, to mention something concerning the tempering of them. It is surprising that this simple process, as simple, almost, as the heating of water, should be so imperfectly understood by so many dentists, and so badly performed by the majority of instrument makers. The easiest method to temper a piece of steel or an instrument is, to make it smooth, or to finish it with the exception of tempering; cover the surface with an alkali, which may be done by dipping it into soft soap, or while hot, into any of the common kinds, then heating to the cherry red, and plunging immediately into moderately cool water. The water may, however, be varied in temperature according to the hardness required. The instrument will then be found clean, of a grayish color, and ready for drawing down the temper to the required tint. The soap prevents the oxidation of the surface; many other substances will answer the same purpose equally well.

It is necessary that points used for packing annealed gold should be quite hard, else in use they will soon lose their sharpness, and at the same time they must be so soft a short distance from the end as not to snap off under pressure. This is effected by holding the point upon a piece of cold steel while the heat is being applied to draw down the temper. It is well to have upon the steel a drop or so of water, so that the end can be suddenly chilled, if the color seems to be running too close. If, after hardening, the steel is polished with lime, or rouge, the slightest changes of color can be observed. Some emery paper or emery cloth may now be used to clean the blackness from the steel above the point, and polished with a common burnisher, when the instrument will be fully ready for use.

These remarks are intended for those of little experience, in forming the points of their instruments. My effort is to present a useful little hint, in a plain manner, to all who may want it.—*Dental News Letter.*

